# The path of ideological and political education in fulfilling the function of psychological nurturing

**DOI:** 10.3389/fpsyg.2023.1202408

**Published:** 2024-04-09

**Authors:** Feng Xue, Naixue Wei, Xinxiong Wu

**Affiliations:** ^1^School of Finance and Economics, Anhui Science and Technology University, Bengbu, China; ^2^College of Information & Network Engineering, Anhui Science and Technology University, Bengbu, China; ^3^Institute for Advanced Studies, University of Malaya, Kuala Lumpur, Malaysia

**Keywords:** ideology, politics, psychological education, structural equation modeling, university students

## Abstract

**Introduction:**

The risk of college students facing psychological problems, such as stress, anxiety, and depression, has increased, which may have a negative impact ontheir academic performance and overall well-being, especially after the outbreakof the pandemic.

**Methods:**

This paper summarizes the potential psychological issues thatuniversity students may face and the corresponding coping measures. Basedon this, a theoretical model of ideological and political education to enhancepsychological education was established.

**Results:**

There was a total of 446 participantsin the study, with a mean age of 21.4 years and 44.6 per cent male. With 406 valid survey responses, the theoretical model was examined using the structuralequation modeling method. The results showed that education and teaching, practical activities, counseling services, prevention and intervention, and multilevelplatforms are effective measures to protect the psychological health ofuniversity students.

**Discussion:**

Based on the insights gained from this study, policies canbe implemented to help university students improve their mental health andinspire higher education institutions to prioritize psychological education.

## 1 Introduction

University students are at an increased risk of experiencing psychological problems, such as stress, anxiety, and depression, which can have negative impacts on their academic performance and overall well-being (Chambel and Curral, 2005; Ram, 2005; Reisbig et al., 2012; Eshraghi et al., 2022). The Coronavirus disease (COVID-19) pandemic has further exacerbated these issues, creating new stressors and challenges for students. The impact of the COVID-19 pandemic on the mental health of university students has been well documented in recent research (Fiorillo and Gorwood, 2020; Stogner et al., 2020; Robinson et al., 2023). Studies have highlighted a range of psychological problems that students are experiencing, including stress, anxiety, depression, loneliness, and social isolation (Tang et al., 2020; Wang et al., 2020; Byrne et al., 2021). One major stressor for university students during the COVID-19 pandemic has been the sudden shift to online learning. Research has found that many students are struggling to adapt to this new mode of learning, which has led to increased levels of stress and anxiety (Dhawan, 2020; Kee, 2021). Furthermore, students have reported feeling overwhelmed by the workload and experiencing difficulties with time management and motivation (Forbus et al., 2011; Biwer et al., 2021). The lack of face-to-face interaction with peers and instructors has also contributed to feelings of isolation and disconnection (Dolan, 2011; Hawkins et al., 2012; Phirangee and Malec, 2020; Singh et al., 2021). The pandemic has also had a significant impact on the mental health of university students due to social isolation. Studies have found that social isolation has led to increased levels of depression and anxiety among university students (Cacioppo and Hawkley, 2003; McLafferty et al., 2021). This has been particularly problematic for students who live alone or who are not able to return home due to travel restrictions. The lack of social support and feelings of loneliness have also been linked to poorer mental health outcomes (Cacioppo and Hawkley, 2003; Luanaigh and Lawlor, 2008; Chen et al., 2014; Wang et al., 2018). Finally, the uncertainty around employment prospects has added another layer of stress for university students during the pandemic. Many students are worried about their future job prospects and are struggling to balance their academic work with job searching (Anwer, 2020; Ashencaen Crabtree et al., 2021). This has led to increased levels of anxiety and worry about the future. Overall, the COVID-19 pandemic has created a range of psychological problems for university students, and it is important for universities to acknowledge and address these challenges and provide support to students during this difficult time.

Researchers have explored potential solutions to these psychological problems, including the use of cognitive-behavioral therapy (CBT) and the promotion of mindfulness and self-compassion practices. CBT has been found to be effective in treating a range of psychological problems, including anxiety and depression (Li et al., 2020; Baghaei et al., 2021). Studies have also found that online CBT can be as effective as in-person therapy for university students (Novella et al., 2022; You et al., 2022). Mindfulness and self-compassion practices have also been shown to reduce stress and anxiety levels and increase well-being (Al-Refae et al., 2021; Yela et al., 2022). Universities have implemented mindfulness and self-compassion programs for students through online platforms (González-García et al., 2021). Universities can also provide additional support and resources for students during the pandemic, including increased access to mental health services, such as online therapy and counseling, and financial support and academic accommodations for students who are struggling to manage their academic workload during the pandemic (Chrikov et al., 2020; Thakur, 2020; Lederer et al., 2021; Liu et al., 2022).

The statistical method known as the structural equation modeling (SEM) is commonly used in psychology to analyze relationships between variables based on their covariance matrix. In psychology and education, certain variables, such as motivation and psychological health, are difficult to measure accurately and are thus referred to as latent variables. The structural equation modeling can handle both these latent variables and their observable indicators, making it a valuable tool for research in these fields. For example, in studies on the impact of COVID-19 on university students’ psychological health and the relationship between mobile phone dependence and psychological health among Chinese undergraduates, the SEM was used to determine significant findings.

In 2017, the Chinese Ministry of Education released the “Implementation Plan for Improving the Quality of Ideological and Political Work in Higher Education Institutions,” outlining the implementation details, paths, and methods of 10 education systems. These systems include curriculum, research, practice, culture, network, psychology, management, service, funding, and organization. The plan emphasizes nurturing the heart and morality, constructing a five-in-one pattern of psychological health education work, and incorporating psychological health education into the overall school curriculum plan. Additionally, the plan proposes establishing a four-level early warning and prevention system, developing guidelines for psychological health education, and cultivating demonstration centers for psychological health education in universities. This paper proposes a theoretical model for ideological and political education to play a psychological nurturing function based on this plan and uses structural equation modeling to verify its path.

## 2 Theories and hypotheses

*H1*: Education and teaching have a significant positive impact on psychological education.

Education and teaching (A) are the fundamental duties and tasks of universities. The achievement motivation theory in the classroom explains that the achievement motivation of teachers and classroom motivation can affect students’ grades and achievements (Damrongpanit, 2019; Şeşen et al., 2019; Kulakow, 2020; Anwar et al., 2021). The application of ethics in counselor education can help students become sensitive to moral issues and improve their reasoning ability, which can help them have the ability to handle psychological problems and think wisely (Pattiwael, 2019; Zeidler et al., 2019; Dewi and Alam, 2020). Teaching can cultivate self-directed learning and critical thinking skills by providing students with opportunities to develop their intuition and logical reasoning abilities, thereby achieving the goal of lifelong education (Costa and Kallick, 2003). Education and teaching play a role in students’ psychological health at different levels and directions, such as cognitive, professional, motivational, personal qualities, and behavioral aspects (Shen et al., 2015; Zee and Koomen, 2016; Weinberg and Gould, 2023).

*H2*: Practical activities have a significant positive impact on psychological education.

Practical activities (B) are an important practical tool for psychological education in higher education. While the acquisition of knowledge and methods in the daily education of students is important, the acquisition of interpersonal relationships and the ability to work with others is a key factor in psychological education (Easterby-Smith, 1997; Martin and Dowson, 2009). Practical activities have a positive impact on students’ interests, and the quality of practical activities is positively correlated with the corresponding student interests (Holstermann et al., 2010). Practical activities have been shown to be highly effective in promoting student learning through collaborative learning, object-based learning and peer interaction that reflects experience (Dabbagh and Kitsantas, 2004). In empirical studies of psychological and behavioral characteristics, the majority of students agree that practical activities are the best way to provide psychoeducation (Pfeiffer, 2013). Practical education can be used as a means of ensuring psychological safety by developing critical thinking, forming a subjective stance, and developing students’ ability to self-manage and level of subjective evaluation (Weiner et al., 2021). Strengthening the practical orientation of education beyond the limits of the “knowledge” educational space develops students’ competence, responsibility, mobility, flexibility, adaptability and competitiveness (Coetzee, 2012).

*H3*: Counseling services have a significant positive impact on psychological education.

Counseling services (C) are a prerequisite for applied psychological education in higher education. Counseling services in European universities are seen as a major resource for student psychoeducation, and counseling systems are more firmly established in Northern Europe than in Southern Europe, even to the extent that counseling is often considered more important than teaching in combination (Yablon, 2020). Counseling can help students with problems in areas such as poor attendance, peer conflict, mental health and coping with stress, which is an important way of improving mental health. Counseling and psychological services need to work in partnership with other student services/support agencies, university administration, curriculum and teaching developers and school staff to ensure that mental health and learning support issues are adequately addressed at all levels (Baruch, 2001). In empirical studies of mental health crises in higher education in North America, counseling centers in educational institutions have struggled to meet the growing needs of students, which has contributed in part to the fact that the number of university students struggling with depression, anxiety, suicidal thoughts and psychosis is on the rise in North America. Resources are needed to increase staffing, improve training and increase physical space in on-campus counseling centers to address the growing number of psychological issues among students (Kitzrow, 2003; Mowbray et al., 2006; Watkins et al., 2012). There is a difference in the need for counseling between undergraduate and graduate students, with the former having a greater need than the latter. In addition, students majoring in science-related fields expressed a greater need than students in the humanities, professional schools, or social sciences (Tidwell and Hanassab, 2007).

*H4*: Prevention and intervention have a significant positive impact on psychological parenting.

Prevention and intervention (D) are core objectives of the psychological nurture function in higher education. Prevention and interventions have been shown to be effective in the prevention and early intervention of depression and anxiety among HE students (Merry and Spence, 2007; Reavley and Jorm, 2010). Preventive mental health interventions in higher education have been shown to have a significant effect in reducing symptoms of depression, anxiety, stress and general psychological distress, as well as in improving social-emotional skills, self-perception, academic behavior and performance (Petrie et al., 2019). Interventions delivered via computers, smartphones or other communication or information devices, rather than primarily face-to-face interventions, have been shown to achieve better outcomes during student mental health interventions (Conley et al., 2016). The need to invest in prevention is supported by the fact that over three-quarters of students with a history of suicidal ideation first developed these thoughts before entering university. The prevention paradigm needs to provide interventions in the student’s environment (e.g., classroom, residence hall, organization) that address the stressors that most often cause distress (e.g., academic stress) and are most distressing (e.g., gender identity concerns, sexual assault) and consider common student coping methods (for better or worse), e.g., drug and alcohol use, spiritual practices. To do this requires a population-centered intervention approach that differs from direct clinical care, particularly for large student populations, and automatically self-renews for future members of the student population (White, 2013).

*H5*: Multi-level platforms have a significant positive impact on psychological education.

The multi-level platform (E) in colleges and universities is a support guarantee for psychological education. The platform guarantee constructed by families, psychological commissioners and counselors is a unique mental health education system in colleges and universities (Le Brocque et al., 2017). Family-school partnerships are beneficial for all students and appear to be particularly important for students whose backgrounds include risk factors such as economic poverty, limited parental education, high family stress and/or cultural discontinuity between home and school (Knoff, 1999). College counselors are the emergency mental health contact for students and mental health staff are the first solution for students facing psychological problems (Kadison and DiGeronimo, 2004). During the COVID-19 pandemic, students’ mental health faced greater challenges, and building a platform for college security was an important support for college students’ mental health (Salimi et al., 2021).

## 3 Empirical analysis

### 3.1 Participants

The formal survey for this study had a total of 446 university students from science and social science departments. The average age of the participants was 21.4 years. The percentage of males among the participants was 44.6%. Participants were 18.3% from the first year of university, 27.9% from the second year, 29.6% from the third year, and 24.2% from the fourth year. The reason for the relatively low percentage of freshmen in this study is that the period of the survey was from September to December, when freshmen were just enrolled in university courses, and the influence of ideological and political education to play the function of psychological nurturing was not necessarily strong. At the same time, the participants in the formal survey came from cities of different sizes. As well as the participants have different economic and educational family backgrounds.

### 3.2 Procedure

In April 2022, based on previous research by scholars, we developed the original survey questionnaire for the theoretical model of the role of ideological and political theory in fostering psychological education. In May and June 2022, we sought feedback from experts in the fields of mental health and structural equation modeling and revised the original questionnaire accordingly. In July 2022, we conducted a pilot study, distributing 20 questionnaires on a small scale. In August 2022, based on the feedback and questionnaire results from the surveyed students, we revised the questionnaire for the second time and created the final version of the survey questionnaire.

### 3.3 Instruments

From September to December 2022, the final version of the questionnaire was distributed to university students through the Questionnaire Star website^1^ and a total of 446 responses were collected, with 406 valid questionnaires and an effective rate of 91.03%, which is to meet the criteria for further research (Mueller, 1999).

### 3.4 Data analysis

#### 3.4.1 Reliability analysis

Reliability, also known as consistency, refers to the degree of consistency in the results obtained when the same object is measured using the same method repeatedly. Cronbach’s alpha coefficient is the most commonly used reliability coefficient, with the formula: $a=\frac{k}{k-1}\left(1-\frac{\sum S_{i}^{2}}{s_{t}^{2}}\right)$. Here, k is the total number of items in the scale, $S_{i}^{2}$ is the within-item variance of the i-th item score, and $s_{t}^{2}$ is the variance of the total score of all items. From the formula, it can be seen that the alpha coefficient evaluates the consistency between the scores of various items in the scale and belongs to the internal consistency coefficient. This method is suitable for reliability analysis of attitude and opinion-based questionnaires (scales).

#### 3.4.2 Validity analysis

Validity analysis usually refers to the validity and accuracy of questionnaire scales, that is, analyzing whether the design of the questionnaire items is reasonable. Validity analysis is divided into exploratory factor analysis and confirmatory factor analysis. Exploratory factor analysis is used to explore the correspondence between factors and measurement items, while confirmatory factor analysis is used to verify the correspondence. For non-classical scales, researchers usually use exploratory factor analysis for validity verification, which is generally called structural validity analysis and is used to demonstrate the validity of the research scale. Exploratory factor analysis of the questionnaire is based on principal component factor analysis, which is achieved by comparing the factor loading coefficients of the items to show the best performance in the same principal component.

#### 3.4.3 Validation factor analysis

The fitting standards of the structural equation model include comprehensive fit index, absolute fit index, and incremental fit index. The comprehensive fit index is used to determine the degree to which the model can predict the covariance matrix and correlation matrix and is evaluated by the CMIN/DF (chi-square degrees of freedom ratio) value. The absolute fit index is used to evaluate the simplicity of the model and is evaluated by the values of RMR (root mean square residual), GFI (goodness of fit index), and AGFI (adjusted goodness of fit index). The incremental fit index compares the theoretical model with the null model and is evaluated by the values of NFI (normed fit index) and TLI (Tucker-Lewis index).

## 4 Results

The reliability coefficient of the overall scale is best above 0.8, acceptable between 0.7 and 0.8, while the reliability coefficient of subscales is best above 0.7, acceptable between 0.6 and 0.7. If Cronbach’s alpha coefficient is below 0.6, it is necessary to consider redeveloping the questionnaire. The results of the data show that the Cronbach’s alpha for all questions from the questionnaire in this study is greater than 0.8, presenting an excellent degree of reliability. Before exploratory factor analysis, KMO test and Bartlett’s sphericity test are performed. The KMO test is used to check the correlation and partial correlation between variables, with values between 0 and 1. The closer the KMO statistic is to 1, the stronger the correlation between variables and the weaker the partial correlation, and the better the effect of factor analysis. In practical analysis, a KMO statistic above 0.7 is generally considered to have a better effect. Detailed data results are shown in Table 1.

**Table 1:** KMO and Bartlett test values.

Kaiser-Meyer-Olkin		0.958
Bartlett’s test of sphericity	Approximate cardinality	13623.823
	Df	378
	Sig	0.000

Using principal component analysis, six principal components were extracted based on eigenvalues greater than one, with a cumulative sum of squared rotated loadings of 82.038%, indicating a more adequate representation of the original data. The detailed results of the validation factor analysis are shown in Table 2.

**Table 2:** Model fit summary.

Fit indicators	CMIN/DF	RMR	GFI	AGFI	NFI	TLI
Value	2.997	0.025	0.865	0.834	0.929	0.944
Standard	1–3	<0.05	>0.80	>0.80	>0.80	>0.80

**Figure 1:**
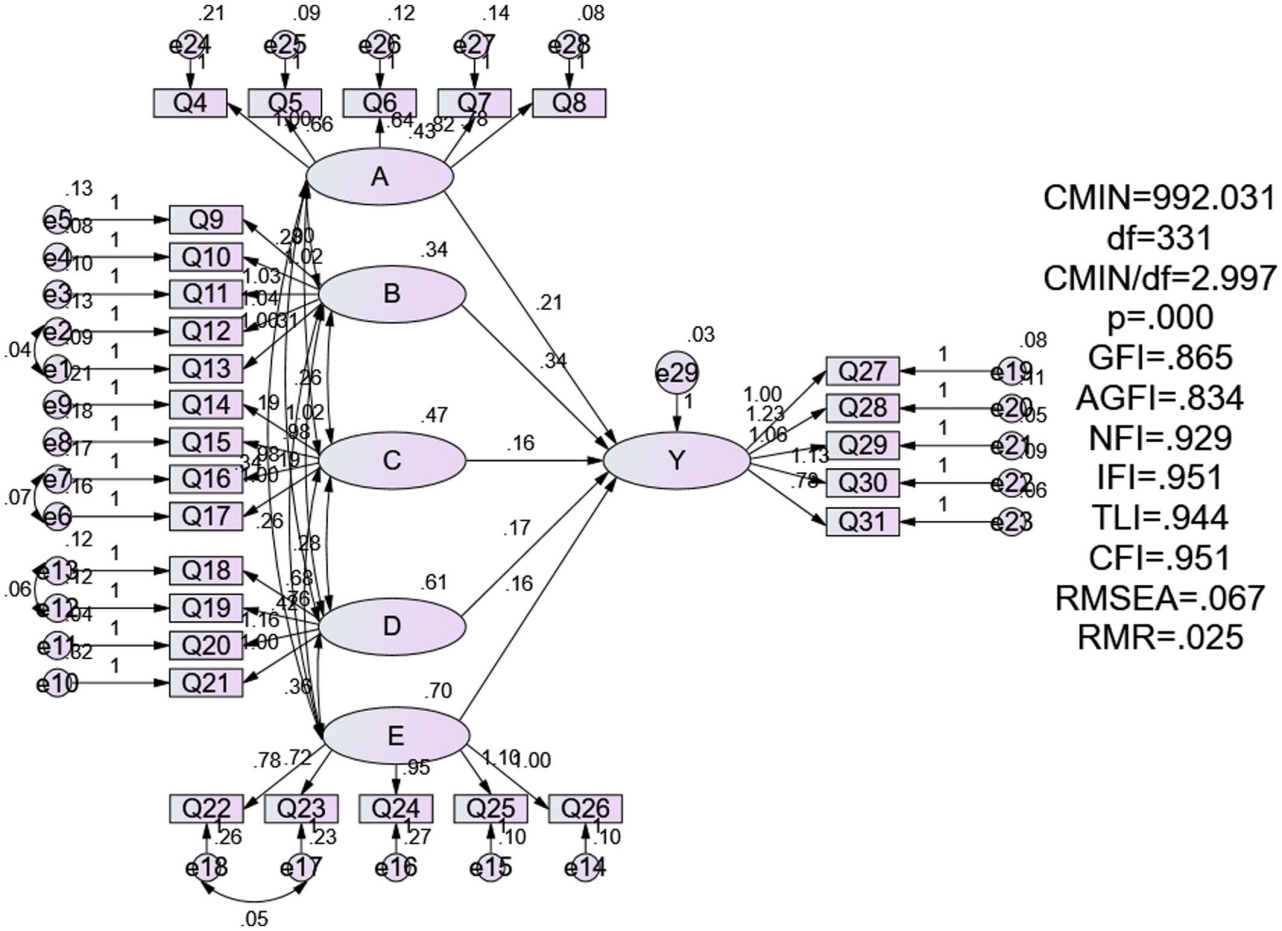
Modified SEM.

Further, the structural equation model is shown in Figure 1.

Table 3 demonstrates the path coefficients and significance level coefficients for the hypothesis test.

**Table 3:** Hypothesis test values.

	Estimate	S.E.	C.R.	*p*
Y←A	0.209	0.033	6.295	***
Y←B	0.336	0.034	9.761	***
Y←C	0.161	0.033	4.926	***
Y←D	0.175	0.019	8.981	***
Y←E	0.156	0.023	6.673	***

When education and teaching influence the mental health of university students, the standardized path coefficient is 0.209 > 0, and this path shows significant significance at the 0.01 level (C.R. = 6.295, *p* = 0.000 < 0.01). Therefore, it can be concluded that education and teaching have a significant positive impact on psychological education, and H1 is validated. Firstly, education and teaching provide students with the knowledge and skills needed to understand and manage their own psychological well-being. This includes learning about stress management, coping mechanisms, and mental health awareness, among other things. By providing students with this knowledge, education and teaching can help them to develop healthy habits and behaviors that support their psychological well-being. Secondly, education and teaching can help to reduce the stigma surrounding mental health issues. By educating students about mental health and encouraging open and honest discussion, education and teaching can help to create a more accepting and supportive environment for students who may be struggling with psychological issues. This can help to reduce feelings of isolation and shame and make it easier for students to seek help when they need it. Finally, education and teaching can help to promote resilience and personal growth in students. By encouraging students to develop their strengths and overcome challenges, education and teaching can help them to develop a sense of purpose and meaning in their lives. This can help to foster resilience and self-esteem and promote positive psychological development over time.

When practical activities influence the mental health of university students, the standardized path coefficient is 0.336 > 0, and this path shows significant significance at the 0.01 level (C.R. = 9.761, *p* = 0.000 < 0.01). Therefore, it can be concluded that practical activities have a significant positive impact on psychological education, and H2 is validated. Practical activities refer to various experiential learning opportunities outside of traditional classroom settings, such as internships, volunteer work, and extracurricular activities. These activities can expose students to real-world situations, help them develop problem-solving skills, and provide opportunities for social interaction and personal growth. In the context of psychological education, practical activities can have a positive impact on students’ mental health by providing them with opportunities to develop self-efficacy, resilience, and a sense of purpose. For example, participating in volunteer work or community service can increase feelings of empathy and social connectedness, while internships or job shadowing experiences can provide a sense of direction and clarity about future career goals. Furthermore, practical activities can provide students with opportunities to apply and integrate the knowledge and skills learned in the classroom, enhancing their overall learning experience. This practical application of knowledge can reinforce students’ understanding of concepts, boost their confidence in their abilities, and increase their motivation to learn.

When counseling services influence the mental health of university students, the standardized path coefficient is 0.161 > 0, and this path shows significant significance at the 0.01 level (C.R. = 4.926, *p* = 0.000 < 0.01). Therefore, it can be concluded that counseling services have a significant positive impact on psychological education, and H3 is validated. Counseling services have a significant positive impact on psychological education because they provide a supportive and safe environment for individuals to explore and address their emotional and psychological challenges. Many students face various personal and academic challenges during their university life, such as stress, anxiety, depression, relationship difficulties, etc. Counseling services can help students to develop better coping skills, problem-solving abilities, and emotional regulation strategies, which can enhance their psychological well-being and academic performance. Moreover, counseling services can also provide individualized support and resources to students, such as referrals to other mental health professionals or community resources, which can further facilitate their personal and academic growth.

When prevention and intervention influence the mental health of university students, the standardized path coefficient is 0.175 > 0, and this path shows significant significance at the 0.01 level (C.R. = 8.981, *p* = 0.000 < 0.01). Therefore, it can be concluded that prevention and intervention have a significant positive impact on psychological education, and H4 is validated. Prevention and intervention can have a significant positive impact on psychological education because they are proactive measures taken to address potential or existing mental health issues before they escalate. By identifying and addressing problems early on, individuals are more likely to receive timely and effective support, which can help to prevent the development of more serious mental health issues. Prevention and intervention can also help individuals develop coping mechanisms and skills to manage stress, anxiety, and other challenges that may arise in their lives, thereby improving their overall psychological well-being. Additionally, prevention and intervention programs can help to reduce stigma surrounding mental health issues and increase awareness and education about mental health, leading to a more supportive and inclusive community.

When multi-level platforms influence the mental health of university students, the standardized path coefficient is 0.156 > 0, and this path shows significant significance at the 0.01 level (C.R. = 6.673, *p* = 0.000 < 0.01). Therefore, it can be concluded that multi-level platforms have a significant positive impact on psychological education, and H5 is validated. Because they provide a comprehensive and diverse range of resources and support for students’ mental health. These platforms can include online resources, peer support groups, counseling services, and institutional policies and practices that promote a healthy and supportive campus environment. By providing students with multiple levels of support, these platforms can help to address a range of mental health concerns and promote positive coping strategies. Additionally, they can help to reduce stigma surrounding mental health and encourage students to seek out help and support when needed. Multi-level platforms can play an important role in promoting psychological education by providing a supportive and inclusive campus environment that prioritizes students’ mental health and well-being.

## 5 Discussion

The results of this study emphasize the considerable and beneficial relationship between education and teaching and the realm of psychological education. Drawing from established theories and principles, these findings provide a solid theoretical foundation to support this assertion. By engaging with education and teaching, individuals not only acquire knowledge but also develop critical thinking, problem-solving skills, and self-efficacy—all of which contribute to their psychological growth (Hyytinen et al., 2018; Simamora and Saragih, 2019). The cognitive development theories of Piaget propose that formal education and teaching provides the scaffolding necessary for individuals to progress through various cognitive stages (Kalina and Powell, 2009; Blake, 2015; Suhendi et al., 2021). This progression not only augments cognitive abilities but also fosters emotional maturity and resilience. Moreover, education and teaching exposes students to diverse perspectives and challenges, nurturing empathy, and emotional intelligence—key components of psychological well-being.

The integration of practical activities into educational contexts has been shown to wield a profound and constructive influence on psychological education. Drawing upon established theories and frameworks, this discussion illuminates the theoretical underpinnings that substantiate the valuable role of practical activities in fostering psychological growth and well-being. Practical activities play a pivotal role in influencing psychological education outcomes. The principles of constructivism and experiential learning, advocated by educational psychologists like Jerome Bruner and David Kolb, emphasize the importance of active engagement and personal experience in the learning process (Bruner and Haste, 2010; Kolb, 2014). These approaches facilitate deeper understanding, emotional engagement, and the development of a growth mindset—factors that significantly contribute to psychological development. Learning is mediated through social interactions, and practical activities often involve collaboration and communication (Potrac et al., 2016). Engaging in group projects or problem-solving tasks nurtures not only cognitive growth but also the development of social skills, emotional regulation, and empathy—integral components of psychological education.

The substantial and affirmative relationship between counseling services and psychological education, buttressed by a solid theoretical underpinning. At the core of the positive impact of counseling services lies the tenets of humanistic psychology, particularly the person-centered approach (Cooper et al., 2013; Joseph, 2021). This approach posits that individuals have an innate drive toward self-actualization and personal growth. Counseling services, guided by this theory, offer a safe and empathetic space for students to explore their thoughts, feelings, and aspirations. Through active listening and unconditional positive regard, counselors facilitate self-exploration and self-acceptance, thereby promoting psychological flourishing.

The integration of prevention and intervention strategies forms a comprehensive framework for nurturing psychological well-being and fostering holistic development. This discussion elucidates the synergistic relationship between these approaches, drawing upon established psychological theories and principles. The transactional model of development, proposed by Sameroff and Chandler, provides a unified theoretical framework for the symbiotic relationship between prevention and intervention (Sameroff, 2010; Biswas et al., 2023). This model suggests that individuals actively shape their environments, and in turn, their environments influence their development. Prevention strategies modify the environment to promote psychological well-being, while intervention strategies, grounded in the same environment, offer targeted support when needed. In conclusion, the convergence of prevention and intervention strategies constitutes a dynamic force in advancing psychological education.

Multi-level platforms that engage families, psychological commissioners, and counselors have emerged as a dynamic force in elevating psychological education, fostering a comprehensive ecosystem of support and growth. This discussion delves into the profound impact of these multi-level collaborations, anchored in theoretical foundations, and their transformative potential in promoting holistic psychological well-being. Bronfenbrenner’s ecological systems theory serves as a cornerstone for understanding the synergy among families, psychological commissioners, and counselors (Sameroff, 2010; Fish and Syed, 2018). This theory emphasizes the interconnectedness of micro and macro systems, illustrating how these multi-level interactions shape an individual’s development (Williams, 2016). The involvement of families, commissioners, and counselors within a cohesive framework aligns with this theory’s emphasis on the intricate interplay between various contexts. Multi-level platforms involving families, psychological commissioners, and counselors signify a transformative approach to psychological education. By aligning diverse stakeholders toward a shared goal of psychological well-being, multi-level platforms empower students to thrive emotionally, academically, and interpersonally. As educational institutions embrace these multi-layered interactions, they sow the seeds for a more resilient and flourishing generation.

## 6 Conclusion

University students’ psychological health faces many challenges, especially after COVID-19. Psychological education is an important part of the ideological and political education system. Based on the experience of previous scholars, this study established a theoretical model of ideological and political education to enhance psychological education. A path investigation questionnaire was designed to investigate the ideological and political education to enhance psychological education. Using the structural equation modeling method, based on the primary survey data of 406 university students, the following conclusions were drawn: Education and teaching, practical activities, counseling services, prevention and intervention, and multi-level platforms have all been found to be important factors in promoting psychological education.

We can protect the psychological health of university students through the following means. Given the challenges faced by university students’ psychological health, it is important to allocate more resources to support psychological education programs on campus. This can include hiring additional counseling staff, increasing the availability of practical activities, and investing in multi-level platforms that promote mental well-being. Incorporate psychological education into the curriculum: Universities can consider incorporating psychological education into the formal curriculum to ensure that all students have access to these resources. This can include classes on stress management, mindfulness, and interpersonal communication. Prevention and early intervention were found to be crucial in promoting psychological education. Universities can consider developing early intervention programs that identify and support at-risk students before their mental health concerns escalate. The study found that counseling services were a critical factor in promoting psychological education. Universities can expand access to these services by offering virtual counseling options, extending counseling hours, and reducing wait times. Finally, universities can foster a culture of well-being by promoting mental health awareness campaigns and initiatives, providing training for faculty and staff on how to support students’ mental health, and creating safe spaces where students can discuss their mental health concerns without stigma or judgment.

## 7 Limitations of the study

This study has provided valuable insights into the impact of ideological and political strategies on psychological education. However, several limitations warrant consideration and suggest directions for future research to enhance the comprehensiveness and robustness of our findings. Future research could employ multi-wave tracking data to examine the causal pathways through which ideological and political factors influence students’ mental health over time, thus overcoming the current limitation. Moreover, the present study’s sample size might restrict the generalizability of its conclusions. To address this limitation, future research could adopt a larger and more diverse sample, encompassing a broader range of cultural, socioeconomic, and demographic backgrounds. The limitations highlighted in this study provide fertile ground for future research endeavors.

## Data availability statement

The raw data supporting the conclusions of this article will be made available by the authors, without undue reservation.

## Ethics statement

The studies involving humans were approved by Academic and Ethics Committee, Anhui Science and Technology University. The studies were conducted in accordance with the local legislation and institutional requirements. The participants provided their written informed consent to participate in this study.

## Author contributions

FX: original manuscript writing. NW: data processing. XW: supervision. All authors contributed to the article and approved the submitted version.

## Footnotes

^1^ www.wjx.cn
